# Supplementary material for: Evidence for genetically-based sperm discrimination in the vaginal tract of a primate species
Source: PLoS Biol. 2026 Mar 31;24(3):e3003699. doi: 10.1371/journal.pbio.3003699 (PMC13038021; doi:10.1371/journal.pbio.3003699)
Supplement: Attachment — Submitted filename: PLOSBio_R1_R2R_final.docx [file pbio.3003699.s010.docx]

Dear Dr. Alvarez-Garcia,

Thank you for your comments and for forwarding the reviews of the two reviewers. We were pleased to see that the reviewers saw this research as addressing an important and poorly-understood question in evolutionary biology. We have addressed their comments regarding the statistical issues inherent to small sample sizes, resulting in a stronger manuscript. If accepted for publication, we would be happy to have our contribution published as a Discovery Report.

Major changes include:

- We now take several additional measures to mitigate the statistical issues inherent to small sample sizes. We refit all models using robust modeling approaches which downweights genes or samples with atypical residual variance. We reanalyzed our gene expression results using empirical Bayes shrinkage and report local false sign rate values (as opposed to p-values), as these methods are more reliable when working with small sample sizes.
- In addition to a multiple hypothesis testing correction across genes, we now also implement a family-wise error rate (FWER) correction per gene across tests and report significant genes as those which pass both an LFSR < 0.1 and FWER-adjusted p < 0.05.
- We now provide effect size estimates with standard errors for all major findings, either within the text for pH analyses or in the Supplementary Tables for gene expression analyses.
- Lastly, we have moderated the claim that this study constitutes the first *in vivo* evidence of sperm discrimination in a primate species throughout the manuscript, clearly stating the limitations due to sample size.

With these new statistical methods, our results remain qualitatively unchanged: we find strong modulating effects of MHC complementarity on postcopulatory vaginal gene expression and pH, suggestive of genetically-based sperm discrimination.

Please find our point by point responses below. Thank you, and please don’t hesitate to contact us with any questions.

Sincerely,

Rachel Petersen and James Higham (on behalf of all authors)

Reviewers' comments

Rev. 1:

The manuscript tackles an important and underexplored question. However, the statistical evidence is fragile due to small sample size, multiple testing, and reliance on interaction models. Results should be interpreted as preliminary, and stronger claims of genetically-based sperm discrimination in primates are not statistically justified at present.

Major Concerns

- The study sample size is very small producing limited dyads. For high-dimensional RNA-seq data, this looks like it is highly underpowered and may lead to increased sensitivity to outliers.

**We agree that our small sample size may increase sensitivity to outliers. To mitigate this issue, we utilized analysis methods appropriate for small sample sizes, including limma-voom count normalization and the use of a blocking term in our models to implement a quasi-paired design. We also characterized how much particular dyads were driving our results by conducting a leave-one-out sensitivity analysis, and found that our results were robust to the exclusion of any particular mating dyad. However, we understand that more could be done to explore the impacts of our small sample size and to mitigate the inherent statistical issues associated with such. We have now taken multiple additional steps.**

1. **We refit our limma–voom model using robust empirical Bayes variance moderation (eBayes(robust = TRUE)), which down-weights genes with atypical residual variance.**
2. **We reanalyzed our model results using empirical shrinkage in the “ashr” package in R, which uses information across genes to shrink effect size and standard error estimates towards 0 and stabilize variance estimate, leading to more stable estimates of both effect size and uncertainty.**
3. **Following effect size and standard error shrinkage, we calculate local false sign rate which estimates the posterior probability that the sign of the effect is wrong. In small sample sizes, the exact p-value or FDR estimate can be noisy, but the overall direction of strong effects is often more reliable than significance itself. This is an especially useful strategy when working with low sample sizes when variance estimates can be very unstable. (Stephens et al. 2017)**

**Stephens, M. (2017). False discovery rates: a new deal. Biostatistics, 18(2), 275-294.**

**Using these updated statistical approaches, our conclusions remain qualitatively unchanged: we continue to observe strong modulatory effects of MHC complementarity on postcopulatory vaginal gene expression. Nonetheless, given the modest sample sizes, the Editor has recommended publication as a *Discovery Report*, a format intended to highlight novel or preliminary findings, and we are pleased to accept this suggestion. We have further revised the language throughout the manuscript to emphasize the preliminary nature of these results and to note that larger sample sizes will be required to confirm and extend our findings. For example:**

***“While these findings are based on a limited dataset and should be interpreted as preliminary, they provide intriguing evidence for a potential mechanism of non-directional sexual selection driven by genetic complementarity (i.e., non-additive mate choice). In this context, genotype-by-genotype interactions would drive CFC, dampening consistent directional shifts in allele frequencies over time across the population. Future work with larger sample sizes will be needed to confirm these patterns, as well as to explore male-driven sexually antagonistic strategies that circumvent female-mediated processes.”* (lines 297-304)**

- Thousands of genes are tested assuming a 10% FDR: how do you justify such a high level? Given small n, variance estimates are unstable, raising the risk of false discoveries.

**We thank the reviewer for raising this point and agree that our explanation of the FDR threshold requires clarification. To generate more robust effect size and uncertainty estimates, we now use “ashr”, which shrinks effect sizes and calculates the local false sign rate (LFSR). LFSR represents the probability that the estimated effect has the correct direction (positive or negative), and is generally more informative than FDR because it incorporates both the magnitude of the effect and the associated uncertainty—rather than relying on p-values alone. This property is especially valuable for studies with modest sample sizes, where uncertainty can be substantial and LFSR appropriately downweights imprecise estimates. An LFSR cutoff of 0.1 (a 10% probability that the estimated effect is in the wrong direction) is widely used, and we now provide references in the text to support this choice. To further demonstrate that our findings are not an artifact of a lenient significance threshold, we now also report how many genes meet more stringent criteria of 5% and 1% LFSR.**

***“We then applied empirical Bayes adaptive shrinkage using the ‘ash’ function from the ‘ashr’ package in R, which borrows information across genes to shrink effect size and uncertainty estimates towards zero, generate more robust posterior estimates, and calculate local false sign rate (LFSR) [43]. LFSR is a measure which integrates both effect size and certainty to generate a posterior probability that an estimated effect is in the correct direction (positive or negative). This approach is particularly advantageous in small-sample settings when variance estimates are unstable because it downweighs imprecise measurements and provides a more reliable alternative to standard FDR corrections [99]. We identified differentially expressed (DE) genes as those falling below a 10% LFSR, a cut-off which is standard in the field of genomics [100-102], and also report the number of DE genes at more stringent 5% and 1% cutoffs.” (lines 555-565)***

***43. Stephens, M., Carbonetto, P., Dai, C., Gerard, D., Lu, M., Sun, L., ... & Zeng, M. (2020). ashr: Methods for adaptive shrinkage, using Empirical Bayes. R package https://github. com/stephens999/ashr. Accessed, 1.***

***99. Stephens, M. (2017). False discovery rates: a new deal. Biostatistics, 18(2), 275-294.***

**100. Resztak, J. A., Wei, J., Zilioli, S., Sendler, E., Alazizi, A., Mair-Meijers, H. E., ... & Pique-Regi, R. (2023). Genetic control of the dynamic transcriptional response to immune stimuli and glucocorticoids at single-cell resolution. *Genome Research,* 33(6), 839-856.**

**101. Lea, A. J., Peng, J., & Ayroles, J. F. (2022). Diverse environmental perturbations reveal the evolution and context-dependency of genetic effects on gene expression levels. *Genome Research*, *32*(10), 1826-1839.**

**102. Petersen, R. M., Vockley, C. M., & Lea, A. J. (2025). Uncovering methylation-dependent genetic effects on regulatory element function in diverse genomes. *Genome Research*, *35*(8), 1781-1793.**

- Several measures of genetic diversity/complementarity are evaluated. Although FDR is controlled within each analysis, no adjustment for the family-wise testing across all these models is provided.

**We thank the reviewer for this suggestion and have now incorporated a family-wise error rate (FWER) correction for each gene across the ten postcopulatory status × genotype models. Specifically, we applied the Holm procedure, which is generally preferred for exploratory analyses, whereas Bonferroni is more appropriate for confirmatory testing (Holm 1979). This additional correction is now described in the Methods, and the Results section has been updated to report the number of genes that meet both our LFSR threshold of 0.1 and the FWER-adjusted p-value threshold of 0.05.**

***“Because we tested genotype × post-copulatory status effects across 10 separate models, we applied an additional family-wise error rate (FWER) correction using the Holm method to adjust the p-values for each gene across all models [106]. In the Results, we report how many genes with LFSR < 10% also meet a FWER-adjusted p < 0.05 after this correction.”* (lines 624-627)**

**106. Holm, S. (1979). A simple sequentially rejective multiple test procedure. *Scandinavian Journal of Statistics*, 6(2), 65-70.**

- Mixed models with few levels for random effects and limited sample sizes are highly instable and reliable variance component estimation is questionable.

**We thank the reviewer for highlighting this limitation. To mitigate this, have used limma-voom with duplicateCorrelation to account for repeated measures while stabilizing variance estimates, and robust empirical Bayes moderation (eBayes(robust=TRUE)) to shrink extreme variances. Additionally, we re-estimated effect sizes and calculated local false sign rates (LFSR) using the R package ‘ashr’, an approach which down weights measures with large error estimates. Our results remain qualitatively the same following these adjustments. Nonetheless, we reiterate across the abstract and discussion that these findings are preliminary and exploratory, and have agreed that this work be published as a *Discovery Report*, a format that supports publication of preliminary findings.**

**Abstract:
*“Our preliminary findings based on six females mating with four males suggest post-copulatory differences in vaginal gene expression and pH, with the strongest immune responses and largest pH decreases, harmful to sperm, exhibited by females mating with genetically similar males.”* (lines 30-33)**

**Discussion:**

***“While these findings are based on a limited dataset and should be interpreted as preliminary, they provide intriguing evidence for a potential mechanism of non-directional sexual selection driven by genetic complementarity (i.e., non-additive mate choice). In this context, genotype-by-genotype interactions would drive CFC, dampening consistent directional shifts in allele frequencies over time across the population. Future work with larger sample sizes will be needed to confirm these patterns, as well as to explore male-driven sexually antagonistic strategies that circumvent female-mediated processes.”* (lines 297-304)**

- Interaction terms between post-copulatory status and genotype are the central findings. Interactions notoriously require even higher sample size to be evaluated compared to main effects, and this study is clearly underpowered. The small sample size makes effect estimates hard to believe.

**We thank the reviewer for this important point. To assess the robustness of our findings in response to this comment, we performed multiple steps to improve our certainty. First, we refitted models with variance stabilizing methods and empirical Bayes moderation, using the lmFit(robust=TRUE) function for gene expression analyses and ‘rlmer’ function in the ‘robustlmm’ packages in R for pH analyses. Second, for our gene expression analyses we use ‘ashr’ to shrink effect size and error estimates and calculate local false sign rate (LFSR), a metric we now use to designate significance instead of FDR, which is calculated using both the effect magnitude and uncertainty rather than using p-values alone. Third, we applied a family-wise error rate correction for each gene across the ten models and report the number of genes that passed an LFSR < 0.1 and a FWER adjusted p-value < 0.05. With these statistical modifications, we find that the interaction effects remain strong.**

***“We performed ten separate differential gene expression analyses, one for each measure of male genetic diversity or complementarity, applying robust estimation and adaptive shrinkage. Each model was constructed with dyad ID as a blocking factor, RIN, cycle phase, and male presence as covariates, and an interactive effect between post-copulatory status (yes or no) and male genotype as the predictor variable. Measures of male MHC diversity and complementarity were associated with an excess of low p-values relative to the null expectation, suggestive that male MHC diversity and complementarity broadly influence gene expression (Fig 3A & B). Genome-wide diversity (stMLH), in contrast, was not as strongly associated with gene expression changes (Fig 3A). Complementarity as measured with MHC I alleles and MHC II alleles was associated with stronger deviations from the null expectation compared to supertypes (Fig 3B). Postcopulatory expression of 456 genes were associated with male MHC allele or supertype diversity, meeting both an LFSR < 0.1 and family-wise error rate (FWER)-adjusted p < 0.05 criteria (S10 Table). Although the different MHC metrics did not share any of the same significant genes, GSEA revealed that male MHC I allelic diversity and MHC II supertype diversity were both positively associated with the expression of genes involved in RNA polymerase activity and intercellular signaling pathways (S12 Table). Likewise, we identified 590 genes whose post-copulatory expression was associated with either MHC or genome-wide complementarity at an LFSR < 0.1 and FWER-adjusted p < 0.05, representing 25 and 17 GSEA pathways, respectively (S11 and S12 Table). Once again, each metric was associated with a unique set of significant genes, however GSEA revealed that both MHC I and MHC II allelic complementarity were both positively associated with pathways involved in immune response and cellular signaling (Fig 3C; S3 and S4 Fig; S12 Table).”* (lines 197-218)**

**Finally, we conducted a leave-one-out sensitivity analysis to test whether the significant interaction effects are dependent on specific mating dyads and present those results in the main text and S6 Fig. Across iterations, we consistently observed a substantial number of DE genes associated with MHC I and MHC II allelic complementarity indicating that this association is not driven by any single dyad. Nonetheless, we continue to present the interaction results as preliminary, replication-ready targets rather than definitive discoveries.**

***“To evaluate the robustness of our findings, we conducted a leave-one-out sensitivity analysis in which we iteratively excluded a single mating dyad from our dataset and re-ran the differential gene expression analysis. For each iteration, we tested the interaction between postcopulatory status and MHC I or MHC II allelic complementarity—two genotype features that yielded the strongest initial associations. Across iterations, we consistently observed a substantial number of DE genes associated with MHC I allelic complementarity (range: 203-1,191, mean = 837.2, SD = 343.1, LFSR < 0.1) indicating that this association is not driven by any single dyad. Differential expression linked to MHC class II allelic complementarity was more variable, yet still consistently present across dyads (range: 86–1,006; mean = 456.3; SD = 317.3, LFSR < 0.1), indicating that our MHC class II results may be more sensitive to the individual dyads included in the analysis. Thus, future studies with larger sample sizes will be necessary to validate these results.”* (lines 238-249)**

- The post-copulatory pH dataset is very limited (15 measurements). Linear mixed modeling with multiple covariates on this dataset is likely over-parameterized.

**We thank the reviewer for pointing this out. To mitigate issues associated with small sample sizes, we have refit our pH models using robust linear mixed effects modeling with the ‘rlmer’ function in the ‘robustlmm’ package in R. Robust linear mixed effects modeling is an extension of standard linear mixed modeling designed to reduce the influence of outliers, non-normal residuals, or extreme random effects. We have also made an effort to simplify models where possible. In our initial models, we included male presence (i.e., whether or not the male had been introduced to the enclosure yet) as a covariate to account for the possibility that unobserved matings occurring more than four hours prior to pH measurement could influence our non-copulatory vaginal pH readings in unknown ways. To evaluate whether male presence meaningfully affected pH in our data, we fit a linear model using only non-copulatory samples and found no evidence of an association (R² = 0.0001, p = 0.859). We therefore believe this covariate can be removed. In addition, we simplified the random-effects structure by replacing separate Female ID and Male ID terms with a single Dyad ID random effect. This approach preserves more degrees of freedom while still appropriately accounting for repeated sampling at the dyad level, which is the relevant unit of analysis. Using this new structure, we now have the power to detect a postcopulatory decrease in vaginal pH (irrespective of male genotype) and have changed the text accordingly:**

***“To assess alterations in vaginal pH following copulation, we used robust linear mixed models including dyad ID as a random effect and cycle phase and average temperature as covariates. Although we did not find an association between cycle phase and pH in our dataset, we included cycle phase as a covariate due to previous work observing lower vaginal pHs around the time of ovulation in baboons and humans [47,48]. We found that post-copulatory pH measurements were significantly lower than non-copulatory measurements (estimate = -0.39, SE = 0.12, p = 0.001; S8 Table; Fig 2E), with substantial individual variation in the magnitude of pH change following copulation (Fig 2F). Although variance tests can be sensitive to small sample sizes, we nonetheless detect significantly greater variance in pH among post-copulatory samples compared to non-copulatory ones (Breusch-Pagan test: p = 0.04), which aligns with our initial hypothesis and supports the use of an interaction model to test for the role of male genetic diversity and complementarity in moderating post-copulatory vaginal pH.”* (lines 168-179)**

**Additionally, we verified the robustness of effect estimates using a leave-one-out sensitivity analysis, in which mating dyads were iteratively excluded to assess if effect sizes remained consistent regardless of the exclusion of any particular dyad. Across all iterations, the direction of the effects of kinship and MHC complementarity on vaginal pH remained consistent, indicating that our findings were not unduly influenced by any individual pairing.**

***“To assess whether our model estimates were driven by particular mating dyads, we refit models testing for the effect of kinship, MHC II allelic complementarity, and MHC II supertype complementarity using the leave-one-out method (see Materials and methods). Our results were generally recapitulated across iterations (S5 Fig). Although smaller sample sizes generate larger standard errors, all model estimates trended in the same direction as the model which included all mating dyads.” (lines 262-267)***

- Variance tests (Breusch-Pagan) with such small samples may not be robust to distributional assumptions.

**We thank the reviewer for pointing this out and now include this caveat in the text.**

***“Although variance tests can be sensitive to small sample sizes, we nonetheless detect significantly greater variance in pH among post-copulatory samples compared to non-copulatory ones (Breusch-Pagan test: p = 0.04), which aligns with our initial hypothesis and supports the use of an interaction model to test for the role of male genetic diversity and complementarity in moderating post-copulatory vaginal pH.” (lines 175-179)***

- Provide effect size estimates with confidence intervals for all major findings (gene expression contrasts, pH changes, genotype interactions).

**We appreciate the reviewer’s suggestion. We have now added these details for our pH analyses in S4, S8 and S14 Tables, and for all differential expression analyses in S2, S6, S10 and S11 Tables.**

Rev. 2:

Overall, we think the statistical methods used in the manuscript are appropriate; the major issue is the sample sizes, or so-called 'large p small n problem'. No matter whether this manuscript will be published or not, we must thank the authors for their efforts and time on the experiments, which are impressive, expansive and exciting to analyze. In the following, not all comments need a response - some are purely for communicating statistical ideas or information; in that case, they are obvious.

**We thank the reviewer for their positive assessment and agree that the limited sample size represents an important constraint of this study. In our initial submission, we implemented several statistical strategies specifically developed for analyses with small sample sizes. For differential expression analyses, we applied limma–voom normalization and incorporated a blocking term to create a quasi-paired design, both of which improve variance estimation and statistical power under small-sample conditions. We additionally conducted a leave-one-out sensitivity analysis, confirming that effect size estimates were stable following the iterative exclusion of individual mating dyads. Nonetheless, we recognize that additional steps could further strengthen the robustness of our inference and we have now taken several new approaches. First, we refit our models using robust estimation approaches, employing robust linear mixed effects models for the pH analyses and robust empirical Bayes variance moderation (eBayes(robust = TRUE)) for the gene expression analyses. These approaches reduce the influence of outliers and genes with atypical residual variance. Second, we reanalyzed model results using empirical shrinkage implemented in the *ashr* package in R, which borrows information across genes to shrink effect size and standard error estimates toward zero, thereby stabilizing variance estimates and improving the reliability of both effect sizes and uncertainty measures. Lastly, we opted to use local false sign rate (LFSR), which estimates the posterior probability that the inferred direction of an effect is incorrect, as opposed to *p* or FDR values to determine significance. In small-sample settings, exact p-values or FDR estimates can be highly variable; however, the direction of stronger effects is often more reliable than nominal significance alone. This framework is therefore particularly well suited to low-sample analyses, where variance estimates can otherwise be unstable. Following these new approaches, our results remain consistent, thus strengthening our findings and interpretation.**

In the 'Genome-wide and MHC genotyping section', we see that the sample sizes (N=13, n=4 males, and n=9 females) are small; when they are divided into subset groups, n is even smaller for each (refer to line 526 for different cycles); so we suggest that the authors would use very conservative languages to communicate their conclusions for the current study and remind the readers that all the conclusions are tentative therefore need larger sample experiments to validate.

**We thank the reviewer for this suggestion. While we have implemented multiple analytical approaches to mitigate statistical challenges associated with small sample sizes, we agree that larger studies will be necessary to fully validate these findings. Accordingly, we now frame our results as preliminary and exploratory both in the abstract and discussion.**

***“Our preliminary findings based on six females mating with four males suggest post-copulatory differences in vaginal gene expression and pH, with the strongest immune responses and largest pH decreases, harmful to sperm, exhibited by females mating with genetically similar males.” (lines 30-33)***

***“While these findings are based on a limited dataset and should be interpreted as preliminary, they provide intriguing evidence for a potential mechanism of non-directional sexual selection driven by genetic complementarity (i.e., non-additive mate choice). In this context, genotype-by-genotype interactions would drive CFC, dampening consistent directional shifts in allele frequencies over time across the population. Future work with larger sample sizes will be needed to confirm these patterns, as well as to explore male-driven sexually antagonistic strategies that circumvent female-mediated processes.” (lines 297-304)***

**Furthermore, due to the preliminary nature of our findings, the Editor has suggested that this manuscript be published as a *Discovery Report*. We are happy to accept this suggestion.**

The word sample is quite confusing throughout the manuscript. We should distinguish between the whole sample size (N as it usually is used in statistics, representing the population that was being studied) and the samples or sample sizes that were a subset of the whole, for example RNA samples (refer to the line 450). So, we'd suggest that the authors add a capital N to the whole sample such as N=13 or whatever is more appropriate in other cases (when N is number of animals, such as N1=9 or N2=4 etc. So that N1+N2+... =13).

**We thank the reviewer for this helpful suggestion. To clarify sample sizes in the text, we now use a capital letter N to denote the full sample, and n with subscript notation to denote subsets of the full sample. For example, when describing the RNA-seq samples included in our postcopulatory analyses they are designated as: N = 62; n_post-cop_= 15, n_non-cop_= 47. We have additionally provided a new supplementary table (S1 Table) which details the number of pH and RNA measurements per female per analysis, with the associated diversity and complementarity measures for each dyad.**

Since N is small, the evidence from the analyses on the results wouldn't be very strong and the validity of the whole study could be greatly reduced, therefore not truly convincing our readers. In other words, if N is smaller than a dozen, then all the statistical models could be invalid, whether it is a mixed model or a simple linear model because there is virtually no degrees of freedom that was left for variation estimation in the model, which will include huge variations, for example, a very wide 95% CI (or large width) on the mean or median value of something measured. Intuitively and statistically approved, it's hard to believe anything deduced from a small sample. Genes, cycles etc. are secondary level variables (derived) in the current study, the first level ones (root, original) are the number of animals, the IDs, the male or female etc., as we generally see from a so-called demographic table (the 1st table usually) in a paper.

**We appreciate the suggestion of a demographic table, and have added a new supplementary table (S1 Table) that details the number of pH and RNA measurements per female per analysis, with the associated diversity and complementarity measures for each dyad. We hope this will enable readers to better evaluate the results in the context of our small sample size. Additionally, we have clarified within the text that our findings are preliminary and that future studies with larger sample sizes will be needed to confirm all results.**

**For example:**

***“While these findings are based on a limited dataset and should be interpreted as preliminary, they provide intriguing support for a potential mechanism of non-directional sexual selection driven by genetic complementarity (i.e., non-additive mate choice). In this context, genotype-by-genotype interactions would drive CFC, dampening consistent directional shifts in allele frequencies over time across the population. Future work with larger sample sizes will be needed to confirm these patterns, as well as to explore male-driven sexually antagonistic strategies that circumvent female-mediated processes.” (lines 297-304)***

On lines 86 to 88, the authors mentioned the number of RNA samples and related number of measurements on those samples, but we'd love to see the number of animals, which is more significant in the current study. And besides, the sample size of the study should be reflected in the ABSTRACT section, where we did not see either. In addition, on line 60 and following lines, we did not observe a description on the sample size of the current study. On lines 84 to 88, we did not observe a description either. On line 176, we should have seen a sample size number, but we did not.

**We thank the reviewer for this suggestion. In addition to our new supplementary table detailing the number of animals, number of samples, and number of sample types included in each analysis (S1 Table), we have also included the number of unique females included within each analysis in the abstract, last paragraph of the introduction, and within each results section.**

**Abstract:**

***“Our preliminary findings based on six females mating with four males suggest post-copulatory differences in vaginal gene expression and pH, with the strongest immune responses and largest pH decreases, harmful to sperm, exhibited by females mating with genetically similar males.”* (lines 30-33)**

**Introduction:**

***“We conducted both genome-wide reduced representation DNA sequencing and MHC genotyping on four intact males and nine parous females and strategically paired each male with 2-3 females to encompass a broad range of genetic diversity and complementarity (i.e., similarity) values across mating dyads.”* (lines 73-76)**

**Results:**

***“We analyzed 32 non-copulatory vaginal RNA samples from eight females (8 per cycle phase) and 275 non-copulatory pH measurements from nine females (68.8 ± 21.8 s.d. per cycle phase; Fig 1A; additional details on dataset composition provided in S1 Table).”* (lines 91-94)

*“We analyzed 25 post-copulatory RNA samples from six females and 15 post-copulatory pH measurements from five females (described in S1 Table), both collected four hours after an observed copulation with ejaculation to maximize potential changes in gene expression [19].”* (lines 133-135)**

Refer to lines 89 and following lines, line 133 and following lines. Since the authors used ID as block factor and it will consume degrees of freedom of the data for each ID included, therefore reduced the effective sample size of the data. How was this issue considered in the processing of the data?

**We thank the reviewer for this thoughtful comment. Including a blocking factor in our model structure was essential due to the repeated measures taken from each female, as it accounts for the non-independence of observations within individuals and prevents pseudoreplication. By modeling ID as a random (or blocking) effect, we capture individual-level variation in baseline expression, thereby improving the accuracy and validity of fixed-effect estimates. In practice, this approach increases model reliability even when degrees of freedom are reduced. Nonetheless, we have edited our interpretation of the findings to be more cautious given the modest sample size (as outlined above) and have agreed to publish this work as a *Discovery Report*, as suggested by the Editor.**

Line 104 and following lines, or line 141 and following lines - the authors used a linear mixed model. How were those variables selected? Any experimental (clinical) or statistical considerations or a purely arbitrary choice?

**We included average temperature as a covariate because animals were housed in non-temperature controlled settings and temperature is known to impact pH readings (Cook et al. 2007, Karlsson & Rosenvold 2002). Although cycle phase was not significantly associated with vaginal pH in our study, previous studies in baboons and humans have observed lower vaginal pH around the time of ovulation, and so we also included cycle phase as a covariate (Miller et al. 2016, Miller et al. 2017). We have now justified these choices in the text.**

***“To assess changes in vaginal pH across the cycle, we used robust linear mixed models [44] including female ID as a random effect and average temperature, which is known to impact pH readings, as a covariate [45, 46].”* (lines 126-128)**

***“To assess alterations in vaginal pH following copulation, we used robust linear mixed models including dyad ID as a random effect and cycle phase and average temperature as covariates. Although we did not find an association between cycle phase and pH in our dataset, we included cycle phase as a covariate due to previous work observing lower vaginal pHs around the time of ovulation in baboons and humans [47, 48].”* (lines 168-172)**

**44. Koller M. Robustlmm: An R package for robust estimation of linear mixed-effects models. J Stat Softw. 2016;75: 1–24.**

**45. Cook, J. D., Strauss, K. A., Caplan, Y. H., LoDico, C. P., & Bush, D. M. (2007). Urine pH: the effects of time and temperature after collection. *Journal of Analytical Toxicology*, *31*(8), 486-496.**

**46. Karlsson, A. H., & Rosenvold, K. (2002). The calibration temperature of pH-glass electrodes: significance for meat quality classification. *Meat Science*, *62*(4), 497-501.**

**47. Miller, E. A., Beasley, D. E., Dunn, R. R., & Archie, E. A. (2016). Lactobacilli dominance and vaginal pH: why is the human vaginal microbiome unique?. *Frontiers in Microbiology*, *7*, 1936.**

**48. Miller, E. A., Livermore, J. A., Alberts, S. C., Tung, J., & Archie, E. A. (2017). Ovarian cycling and reproductive state shape the vaginal microbiota in wild baboons. *Microbiome*, *5*(1), 8.**

On line 196, we see a very tiny p value, but this comes from a very small sample size of study, which is a very common dilemma in a genetic study. Statistically, a very small p value should come from a large sample. And a common misunderstanding about the p value is that the statistical null hypothesis is that there is no difference or difference is zero, a p value only tells us that the difference is different from zero (not zero), but how different is not solved. For example, the difference of 0.002 is different from zero, and 0.02 or 0.2 is different from zero but they are 10 or 100 times larger or smaller comparing one to another.

**We agree that information on the strength of effect is important here, and we now include effect size estimates and standard errors for all of our results either in the text or in the Supplemental Tables.**

On line 211, the numbers look reasonable, but on line 214, we see a different scenario: the range is huge, because this is small sample size, and anything derived from a small sample would be an unstable sample, does not follow a normal distribution (when median is close to the mean value), which is why people have to rely upon a median value (like median income); however, a median is farther away from the truth (the mean is better, at least a trend, the characteristic or the majority of something). So, we need a large sample to validate a conclusion generally.

**We agree and have added text to this section articulating the need for a larger sample size to validate these findings.**

***“Thus, future studies with larger sample sizes will be necessary to validate these results.” (lines 248-249)***

In Table 1 and in Figure 3, again, we don't know the sample sizes, so we are not convinced that the results are good to generalize to other cases or external data, this is, we are not certain whether we should accept the conclusions.

**We thank the reviewer for pointing this out. We have now reiterated our sample size in the legends of Table 1 (now S13 Table) and Fig 3.**

***“S13 Table. Number of genes/pathways whose post-copulatory expression is significantly modified by an aspect of male genetic makeup across five mating dyads”***

***“Fig 3. Post-copulatory vaginal gene expression in relation to male diversity and complementarity in five mating dyads”***

In Figure S5, we are not sure what that "p < 0.05" is, please add an explanation at the footnote.

**This legend was meant to signify whether an effect was statistically significant or not. We have changed the legend to instead read “significant” and clarify this in the figure legend.**

**“*The genotype included in each model is indicated in the facet title and the color designates whether or not the interaction term in the model was significant (LFSR < 0.1)*”**

**We hope that with the above responses and changes to the manuscript, it might now be considered acceptable as a *Discovery Report* in PLoS Biology. Thank you for considering our revision for publication.**

**Rachel Petersen, and all coauthors.**
