# Supplementary material for: Evidence for genetically-based sperm discrimination in the vaginal tract of a primate species
Source: PLoS Biol. 2026 Mar 31;24(3):e3003699. doi: 10.1371/journal.pbio.3003699 (PMC13038021; doi:10.1371/journal.pbio.3003699)
Supplement: Attachment — Submitted filename: PLOSBio_R2_R2R.docx [file pbio.3003699.s011.docx]

February 20, 2026

Dear Dr. Alvarez-Garcia,

Thank you very much for the positive update. We are delighted that the reviewers found the manuscript improved and suitable for publication, and we appreciate the opportunity to provide the additional requested information.  Please see our responses to requests below.

- We have now included as Supplementary files (S1-S5 Data) all of the dataframes necessary to generate figures. We reference within each figure legend the data file needed to generate all panels of that figure.
- We provide on Zenodo the input data for all analyses and figures (<https://zenodo.org/records/14976902>) and the current version of our github repository (<https://zenodo.org/records/18705035>)
- We have updated the Data Statement to reflect the additional Supplementary files (S1-S5 Data) and the Zenodo record containing the github code repository.
- We have added reference to the study species (olive baboon; *Papio anubis*) in the abstract.

Thank you for making a suggestion for an alternative title. We deliberately tried to use words to describe what we had observed, and avoided using words that implied we thought that there was necessarily female agency or invoked ideas surrounding top-down cognitive control of the process. We are concerned that the language of “A female primate *can exert*..” indeed implies such agency. For these reasons, we would prefer to retain our original title:

“Evidence for genetically-based sperm discrimination in the vaginal tract of a primate species”

Alternatively, we would be happy to revert to the original title submitted to *Science*, which was previously suggested for revision:

“Mechanisms of cryptic female choice in the vaginal tract of a primate species.”

We believe either of these options more accurately reflects the content and tone of the manuscript while remaining clear and accessible to readers.

Thank you again for your guidance throughout the review process. Please do not hesitate to let us know if any further clarification is needed.

Sincerely,
Rachel Petersen and James Higham
(on behalf of all authors)
